# Neuronal Damage Induced by Gradual Oxidative Stress in iPSC‐Derived Neurons: Implications for Ferroptosis Involvement and ALS Drug Evaluation

**DOI:** 10.1111/jnc.70246

**Published:** 2025-09-28

**Authors:** Hayato Kobayashi, Hitoshi Suzuki‐Masuyama, Hirokazu Tanabe, Hiroshi Kato, Setsu Endoh‐Yamagami

**Affiliations:** ^1^ Bio Science & Engineering Laboratories FUJIFILM Corporation Kanagawa Japan

**Keywords:** ALS, cholesterol, edaravone, ferroptosis, motor neurons, oxidative stress

## Abstract

The molecular mechanisms underlying neurodegenerative diseases are not fully understood, but oxidative stress is known to play a central role in the pathogenesis of neurodegenerative diseases, including amyotrophic lateral sclerosis (ALS) and Alzheimer's disease (AD). In this study, we developed a method to induce gradual oxidative stress in induced pluripotent stem cell (iPSC)‐derived motor neurons and cortical excitatory neurons by omitting antioxidants in the media, aiming to create a platform for studying oxidative stress‐dependent neuronal damage in neurodegenerative diseases. Neuroprotective effects in this platform were observed with edaravone, an approved ALS medicine, in iPSC‐derived motor neurons, suggesting its potential for ALS drug evaluation. The oxidative stress‐induced neuronal damage was accompanied by increased lipid peroxidation, and it was suppressed by ferroptosis inhibitors and an iron‐specific chelator, suggesting that neurons died through ferroptosis. Furthermore, through a compound screen, a cholesterol biosynthesis inhibitor, AY 9944, was identified as being capable of inhibiting neuronal damage induced by oxidative stress. Additionally, neuroprotective activity was observed with 7‐dehydrocholesterol, an immediate precursor of cholesterol, while the efficacy of AY 9944 was compromised by knockout of the *EBP* gene, which encodes an enzyme involved in cholesterol biosynthesis. These findings suggest the involvement of ferroptosis in the progression of neurodegenerative diseases and the inhibition of ferroptosis by modulating the cholesterol biosynthesis pathway, providing potential insights for drug development.

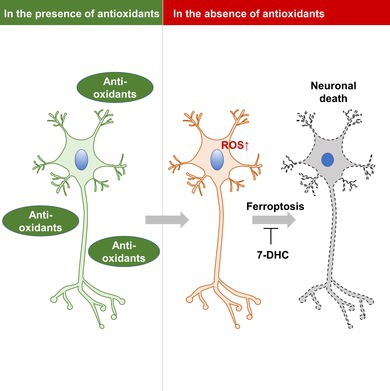

## Graphical abstract

The authors induced damage in iPSC‐derived motor neurons through gradual oxidative stress by culturing them without antioxidants. Neurons underwent ferroptosis‐dependent cell death, which was inhibited by edaravone. Additionally, the authors revealed neuroprotective activity in a cholesterol metabolite and a sterol pathway inhibitor, suggesting that modulation of the cholesterol biosynthesis pathway protects neurons from oxidative stress‐induced neuronal damage.

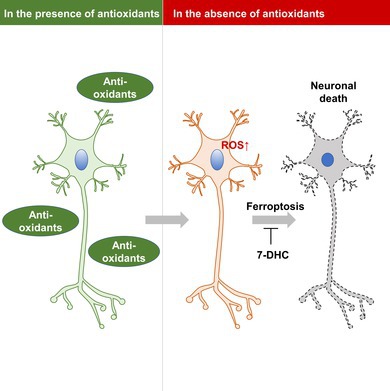

**Abbreviations**
7‐DHC: 7‐dehydrocholesterol
AD: Alzheimer's disease
ALS: amyotrophic lateral sclerosis
AO: antioxidants
AUC: area under the curve
DHCR7: 7‐dehydrocholesterol reductase
GWAS: genome‐wide association study
iPSC: induced pluripotent stem cell
ROS: reactive oxygen species

## 1 Introduction

Neurodegenerative diseases are chronic progressive disorders characterized by the gradual damage of neurons in the nervous system, including amyotrophic lateral sclerosis (ALS) and Alzheimer's disease (AD). The molecular mechanisms of neurodegenerative diseases are not fully understood, but a substantial body of research suggests that oxidative stress, induced by both genetic and nongenetic risk factors, constitutes one of the central mechanisms underlying ALS and AD pathogenesis (Mead et al. 2023; Barber and Shaw 2010; Cunha‐Oliveira et al. 2020; Hemerková and Vališ 2021; Nunomura et al. 2006). Genome‐wide association studies (GWAS) of ALS and AD revealed greater involvement of genetic factors in the diseases than previously recognized (van Rheenen et al. 2021; Bellenguez et al. 2022). The risk variants, however, have a limited effect on survival and age of onset (van Rheenen et al. 2021; Bellenguez et al. 2022). The GWAS analyses and other studies indicate that ALS is a complex disease caused by a combination of multiple genetic and nongenetic risk factors (van Rheenen et al. 2021; Al‐Chalabi et al. 2014; Duan et al. 2023; Mead et al. 2023). The nongenetic risk factors can be affected by lifestyle and environment, and they can explain the adult onset of ALS (Al‐Chalabi et al. 2014; Duan et al. 2023; Mead et al. 2023). Among the pathogenesis triggered by both nongenetic and genetic factors, we focused on oxidative stress.

Oxidative stress arises from an imbalance between the production of reactive oxygen species (ROS) and the ability to detoxify ROS in cells. Oxidative stress leads to damage to cellular structures such as membranes, lipids, proteins, lipoproteins, and DNA (Pizzino et al. 2017). Increased oxidative stress biomarkers in neurodegenerative disease patients, including ALS and AD, have been reported (Mitsumoto et al. 2008; Blasco et al. 2017; Cioffi et al. 2021). Oxidative stress interacts with various processes implicated in the onset and progression of both ALS and AD (Barber and Shaw 2010; Bai et al. 2022), including excitotoxicity, mitochondrial dysfunction, protein aggregation, cytoskeletal dysfunction, and glial dysfunction. Consequently, oxidative stress is believed to play a role in neuronal cell death.

The mechanism of neuron death in neurodegenerative diseases remains controversial, but ferroptosis is attracting attention as it is implicated in various diseases, including ALS and AD (Ou et al. 2022; Wang et al. 2023; Wang, Wang, et al. 2022; Wang, Tomas, et al. 2022; Jakaria et al. 2021). Ferroptosis is a recently discovered form of iron‐dependent programmed cell death characterized by the accumulation of lipid peroxides, which distinguishes it from apoptosis, necrosis, necroptosis, and other forms of cell death (Dixon et al. 2012; Jiang et al. 2021). ALS patients have been reported to exhibit ferroptosis‐related features, such as increased lipid peroxidation, along with elevated markers of DNA oxidation and neuronal injury in their plasma samples (Devos et al. 2019). More recently, genome‐wide CRISPR screening from three independent groups revealed the involvement of 7‐dehydrocholesterol reductase (DHCR7) in ferroptosis, leading to the identification of 7‐dehydrocholesterol (7‐DHC), an intermediate metabolite of cholesterol biosynthesis, as an endogenous anti‐ferroptotic metabolite particularly in cancer and organ ischemia–reperfusion injury (Freitas et al. 2024; Li et al. 2024; Yamada et al. 2024). However, the role of the cholesterol pathway has not been elucidated in neurodegenerative diseases in relation to ferroptosis.

There are high expectations for in vitro human disease models to enhance our understanding of disease mechanisms and improve clinical prediction, ultimately increasing the success rate of drug development. Drug development for ALS is active, with over 60 compounds with different mechanisms of action being investigated in clinical trials (Mead et al. 2023), in addition to the FDA‐approved drugs, such as riluzole, edaravone, and tofersen. Human induced pluripotent stem cell (iPSC) technology has allowed for the generation of human cell‐based models, leading to the reporting of numerous iPSC models carrying ALS‐related gene mutations (Giacomelli et al. 2022). A noteworthy study utilizing genome‐wide CRISPR interference and activation screens in human iPSC‐derived neurons has shed light on the role of the lysosomal protein prosaposin gene. Knockdown of this gene sensitizes neurons to oxidative stress and triggers ferroptosis, providing deeper insights into the mechanisms underlying neurodegenerative diseases (Tian et al. 2021). On the other hand, a drug evaluation system that is not limited to specific gene mutations but is centered on oxidative stress and can be widely used as a neuronal damage model for diseases without specific mutations would be useful for therapeutic drug development.

In this study, we induced neuronal damage in human iPSC‐derived cortical neurons and motor neurons originating from healthy donors by culturing neurons in media lacking antioxidants to induce gradual oxidative stress. The neurons exposed to oxidative stress exhibited increased cell death and elevated cellular ROS levels. The neuronal damage was inhibited by edaravone, suggesting its potential use as an ALS model. Additionally, the neuronal damage was suppressed by ferroptosis inhibitors and an iron‐specific chelator, accompanied by increased lipid peroxidation, suggesting that neurons died through ferroptosis in the oxidative stress disease model. Through a compound screen, AY 9944, a distal cholesterol biosynthesis inhibitor, was identified as a protective agent against neuronal damage caused by oxidative stress. Furthermore, our model revealed that 7‐DHC exhibited robust activity in protecting neurons from oxidative stress‐induced damage. These findings demonstrate that the neuronal damage model established in this study will be useful for developing ALS therapeutics and understanding the molecular mechanisms underlying ALS and other neurodegenerative diseases.

## 2 Materials and Methods

### 2.1 Neurons

Human iPSC‐derived motor neurons were purchased from FUJIFILM Cellular Dynamics (iCell Motor Neurons‐01279, cat. no. C1048) and Axol Bioscience (axoCells Human iPSC‐Derived Motor Neurons, cat. no. AX0078). iCell Motor neurons were primarily used as motor neurons, unless otherwise specified. FF‐1 and FF‐2 iNeurons, excitatory cortical neuron‐like cells, were generated by inducing NGN2 expression in the FF‐1 and FF‐2 human iPSC lines (provided by FUJIFILM Cellular Dynamics), respectively. A clonal iPS cell line with a doxycycline‐inducible mouse *Ngn2* transgene inserted at the AAVS1 locus was established using the AAVS1 Safe Harbor Targeting System (System Biosciences). These iPSCs were then differentiated into iNeurons by doxycycline treatment as described (Zhang et al. 2013). FF‐1 iNeurons were generated within three passages after confirming the normal karyotype at the FF‐1 iN‐iPSCs stage, which are iPSCs carrying the NGN2 expression cassette. FF‐2 iNeurons were generated within seven passages from FF‐2 iPSCs following karyotype confirmation. The expression of glutamatergic excitatory neuron markers (*VGLUT1/VGLUT2* mRNA and VGLUT1 protein) and a motor neuron marker (*CHAT* mRNA and ChAT protein) was confirmed in FF‐1 and FF‐2 iNeurons and iCell Motor neurons, respectively, under the specified assay conditions of culturing in DMEM/F12 with antioxidants for 3–4 days (Figure S1).

The *EBP* homozygous knockout (*EBP* ^−/−^) neurons were generated by introducing sgRNA targeting the *EBP* gene (Thermo Fisher Scientific) and Alt‐R S.p. HiFi Cas9 nuclease V3 (Integrated DNA Technologies), followed by single clone isolation using the iPS cell line (P020_C9_02_08) carrying DOX‐inducible NGN2 expression cassettes. This iPS cell line was established from a sporadic Alzheimer's disease patient (Kondo et al. 2022) through a previous collaboration between Kyoto University and FUJIFILM Toyama Chemical, and the characterization of the patient iPSC‐derived neurons was reported elsewhere (Kondo et al. 2022). The *EBP* targeting sequence is described in Figure 4e. The cells used in this study are not listed by ICLAC as commonly misidentified. We have maintained records of the cell culture process from receipt, thawing, culturing, and expansion to create stocks, but we did not conduct authentication.

### 2.2 Neuronal Culture

For the condition without oxidative stress (+AO), neurons were cultured in DMEM/F12 (Thermo Fisher Scientific, 11320033) with B‐27 supplement (50×), serum free (Thermo Fisher Scientific, cat. no. 17504‐044). For the condition with oxidative stress (−AO), neurons were cultured in DMEM/F12 with B‐27 supplement (50×), minus antioxidants (Thermo Fisher Scientific, cat. no. 10889‐038). Motor neurons were plated at 3500 cells/well of a 384‐well plate or 15 000 cells/well of a 96‐well plate. The poly‐d‐lysine‐treated 384‐well plates (PerkinElmer, cat. no. 6007710) or 96‐well plates (Corning, cat. no. 356461 or 356640) were coated with iMatrix‐511 silk (Matrixome, cat. no. 892021) before plating the neurons. FF‐1 and FF‐2 iNeurons were cultured in DMEM/F12 or neurobasal media (Thermo Fisher Scientific, cat. no. 21103049) with B‐27 supplement (50×), serum free or B‐27 supplement (50×), minus antioxidants. FF‐1 iNeurons were plated at 15 000–30 000 cells/well, and FF‐2 iNeurons were plated at 15 000 cells/well in a 96‐well plate. The *EBP* ^−/−^ neurons and their control (*EBP* ^+/+^) neurons were plated at 15 000 cells/well in a 96‐well plate, with or without supplementation of 1.5 μg/mL cholesterol.

### 2.3 Compounds and Treatment

The neurons were cultured in the presence of DMSO up to 0.1% unless otherwise described, and it was confirmed that this concentration did not affect neurite lengths (data not shown). Compounds other than sterols were dissolved in DMSO and added to the culture medium: edaravone (FUJIFILM Wako Pure Chemical), riluzole (MedChemExpress), Z‐VAD‐FMK (Adipogen), necrostatin‐1 (ChemScene), necrosulfonamide (Cayman Chemical), ferrostatin‐1 (MedChemExpress), liproxstatin‐1 (MedChemExpress), GSK‐872 (MedChemExpress), UAMC‐3203 hydrochloride (MedChemExpress), RIPA‐56 (MedChemExpress), deferoxamine (MedChemExpress), and AY 9944 (Cayman Chemical). Stock solutions of 7‐dehydrocholesterol (Merck), lathosterol (Merck), and cholesterol (Avanti Polar Lipids) were prepared using ethanol, and they were applied to neurons at a final concentration of 0.1% ethanol.

For the compound screen, the StemSelect Small Molecule Regulators Library (Merck), containing 303 pharmacologically active small molecules, was used with a 10 000‐fold dilution (final concentration of 1 μmol/L, with some exceptions).

### 2.4 Neurite Length Analysis

For neurite length analysis, time‐lapse images were captured, and the total neurite length (mm/mm^2^) was measured every 6 h using the IncuCyte S3 Live‐Cell Analysis System with the NeuroTrack Software module (Sartorius), from the start of measurement until 13 days (312 h, 53 time points), except for screening cultured for 14 days. Two and four field images were captured per well in 384‐well and 96‐well plates, respectively. Wells exhibiting noticeable abnormalities, such as plate scars, were excluded from the data analysis. The area under the curve (AUC) for total neurite length measurement was calculated for each compound treatment to evaluate the neuroprotective effects. The maximum total neurite length of each well was determined. Subsequently, the MAX values of the −AO control group were averaged, and half of this average was defined as 50% MAX of the control. H50 was calculated as the point of time when the total neurite length became smaller than 50% MAX of the control for the first time after observation of MAX. In the case if the total neurite length did not decrease below the 50% MAX of the control by the end of measurement, the H50 was set to 312 h.

### 2.5 LDH Cytotoxicity Assay and ROS Detection

LDH cytotoxicity assays were performed using Cytotoxicity Detection Kit^PLUS^ (LDH) (Merck), and the absorbance at 490 nm was measured using 640 nm as a reference. To measure cellular ROS levels, CellROX Green Reagent (Thermo Fisher Scientific) was used following the manufacturer's protocol. After incubation with CellROX Green Reagent, cells were fixed with 3.7% formaldehyde followed by Hoechst 33342 (DOJINDO) nuclear staining. The mean intensity of the CellROX Green signal in the nuclei was measured using the CellVoyager CQ1 Benchtop High‐Content Analysis System (Yokogawa).

### 2.6 Lipid Peroxidation Assay

To measure lipid peroxidation in cell membranes, BODIPY 581/591 C11 (Lipid Peroxidation Sensor; Thermo Fisher Scientific) was used according to the vendor's protocol. Neurons were treated with BODIPY 581/591 C11 and collected from culture plates using TrypLE Select Enzyme (Thermo Fisher Scientific). The collected cells were stained with Fixable Viability Stain 780 (BD Biosciences) and analyzed by flow cytometry (Attune NxT Flow Cytometer, Thermo Fisher Scientific) to measure the green fluorescent signal in live cells. The data were visualized using FlowJo v10.10 (BD Biosciences).

### 2.7 Statistical Analysis

Statistical analysis was performed using GraphPad Prism 10 software. For experiments comparing two groups, a two‐tailed unpaired Student's *t*‐test was performed. To compare multiple groups, two‐way ANOVA followed by Bonferroni's or Dunnett's multiple comparison test was conducted, as specified in the figure legends or notes of Tables S1–S14. The significance level was set at 5% with two‐tailed probability. The normality tests were performed using the Shapiro–Wilk test for input data of the LDH and ROS assays, as well as the control (−AO condition) data of neurite length assays in motor neurons, FF‐1 iNeurons, and FF‐2 iNeurons, utilizing the data sets from Tables S1, S2, and S11–S14. No statistical testing for outliers was performed.

## 3 Results

### 3.1 Induction of Neuronal Damage by Oxidative Stress

To establish an in vitro assay platform that reflects oxidative stress in neurodegenerative diseases using neurons without known disease‐related mutations, we cultured iPSC‐derived neurons from healthy donors and exposed them to gradual oxidative stress by omitting antioxidants (AO) from the culture medium, utilizing a commercially available B27‐supplement minus AO. Typically, neurons are cultured in the presence of AO, such as vitamin E, vitamin E acetate, superoxide dismutase, catalase, and glutathione. Neurobasal medium, a modified DMEM/F12 that lacks ferrous sulfate and the excitatory amino acids glutamate and aspartate, is preferred for promoting better survival of neurons in culture (Brewer et al. 1994). Neurons are usually cultured at high density because neurons seem to express endogenous growth factors (Lowenstein and Arsenault 1996).

**FIGURE 1:**
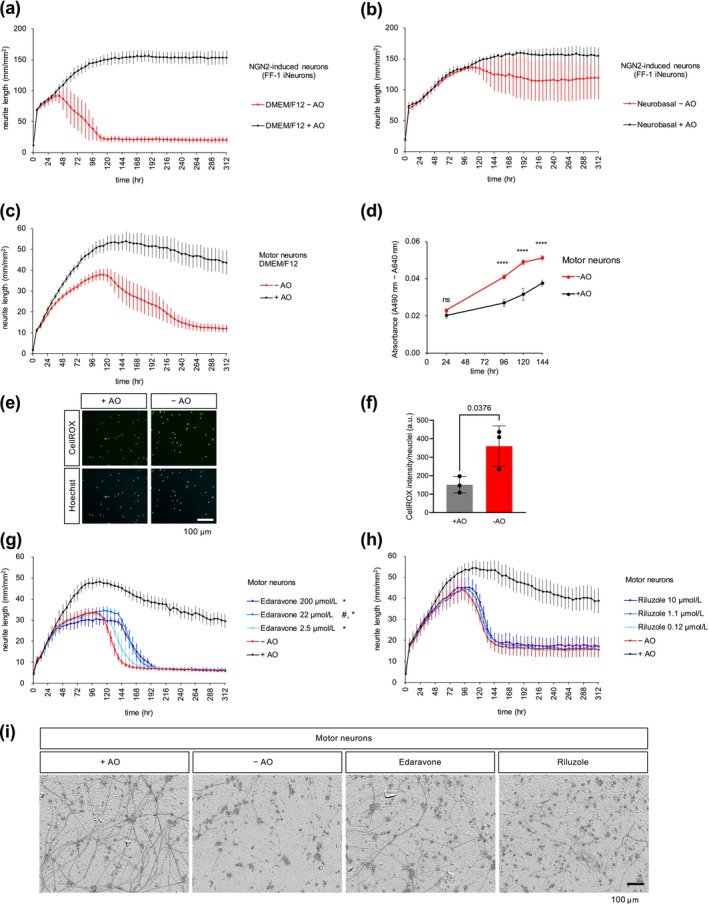
Neuronal damage induced by oxidative stress. (a) Time course changes of the total neurite length (mm/mm^2^) of NGN2‐induced neurons, FF‐1 iNeurons, cultured in DMEM/F12 with antioxidants (AO): +AO or without AO: −AO. The lines represent the average of three experiments using cells prepared independently. Error bars show standard error of the mean (SEM). Each experiment consists of 2–6 wells per condition. (b) Time course changes of the total neurite length (mm/mm^2^) of FF‐1 iNeurons cultured in Neurobasal with AO (+AO) or without AO (−AO). The lines represent the average of three experiments using cells prepared independently. Error bars show standard error of the mean (SEM). Each experiment consists of 2–6 wells per condition. (c) Time course changes in the total neurite length (mm/mm^2^) of motor neurons (iCell Motor Neurons‐01279; FUJIFILM Cellular Dynamics) cultured in DMEM/F12 with AO (+AO) or without AO (−AO). The lines represent the average of eight experiments using different lots of motor neurons. Error bars show SEM. Each experiment consists of 4–14 wells per condition. (d) Time course measurement of the LDH levels in the medium of motor neurons cultured in DMEM/F‐12 with AO (+AO) or without AO (−AO). The LDH levels were calculated as the average of three wells in each condition. Error bars show the standard deviation. Statistical analysis was performed by two‐way ANOVA followed by Bonferroni's multiple comparison test; ns: *p* = 0.2882, ****P < 0.0001. (e, f) ROS detection and quantification using CellROX Green Reagent at Day 6 (around 144 h) of motor neurons in the +AO and −AO conditions. Scale bar: 100 μm. Mean signal intensity in the nuclei was quantified. The average of three wells (more than 450 cells in each well) and standard deviation are shown. Statistical analysis was performed using a two‐tailed unpaired Student's *t*‐test. (g, h) Time course changes in the total neurite length of motor neurons cultured in media without AO in the presence of edaravone or riluzole. The lines represent the average of three and four experiments with independent cell culture preparations for edaravone and riluzole, respectively. Error bars indicate SEM. Each experiment consists of 13–14 wells for the −AO and +AO groups, and four wells for compound treatment in (g), as well as 6–14 wells for the −AO and +AO groups, and 2–4 wells for compound treatment in (h). Significant effects increasing AUC and H50 are indicated by # and *, respectively, as determined using two‐way ANOVA followed by Dunnett's multiple comparison test (^#^ *p* = 0.0013 at 22 μmol/L; *p = 0.0001 at 2.5 μmol/L and *p* < 0.0001 at 22 and 200 μmol/L). See Tables S1–S4 for detailed statistical analysis. (i) Representative images of motor neurons at 168 h. The concentrations of edaravone and riluzole are 200 and 10 μmol/L, respectively. Scale bar: 100 μm.

We tested various media and cell densities and identified a condition that caused neuronal damage when combined with the removal of AO (Figure 1). When NGN2‐induced excitatory cortical neurons were cultured in DMEM/F12 medium with B27 supplement minus antioxidants (AO) at a relatively low density (30 000–100 000 cells/mm^2^), they initially extended neurites, but a reduction in neurite length was observed after a certain period (Figure 1a). In contrast, neurons cultured in the medium with B‐27 supplement containing AO extended neurites for a longer duration (Figure 1a). On the other hand, neurons cultured in Neurobasal medium extended and retained neurites even in the absence of AO (Figure 1b). We considered this absence of antioxidants in DMEM/F12 medium to mimic a chronic oxidative stress condition, where neurons initially survive and extend neurites through the cellular antioxidant defense system but eventually become unable to tolerate the oxidative stress, leading to reduction in neurite length.

To examine if neuronal damage is also induced in iPSC‐derived motor neurons, we cultured motor neurons (iCell Motor neurons) in the absence of AO in DMEM/F12 medium (Figure 1c). Motor neurons cultured in the absence of AO initially extended neurites, but subsequently began to show signs of damage as well (Figure 1c, see representative images of neurons in Figure 1i).

To confirm that the neuronal damage is accompanied by cell death, we performed a lactate dehydrogenase (LDH) cytotoxicity assay. The levels of LDH released in the medium were higher under oxidative stress compared to the absence of stress, indicating increased cell death under oxidative stress conditions (Figure 1d). The elevated ROS levels in the motor neurons under oxidative stress were also confirmed using CellROX Green, a fluorogenic probe for ROS (Figure 1e,f).

To examine whether approved drugs for ALS could protect neurons from damage, motor neurons were incubated with edaravone or riluzole under oxidative stress (Figure 1g,h). The area under the curve (AUC) for total neurite length measurement was calculated for each compound treatment. Motor neurons cultured in DMEM/F12 without AO (−AO) exhibited an AUC of 4781 ± 358 mm/mm^2^ h (mean ± SEM, *n* = 3 experiments with independently cultured cells). Edaravone significantly increased the AUC to 5586 ± 328 at 22 μmol/L compared to the −AO condition (Table S1). The ability of compounds to delay neuronal damage was also assessed by calculating H50 (h) as the time when total neurite length decreased to < 50% of the maximum total neurite length in the −AO control. Neurons under the −AO condition had H50 of 133 ± 5 h (mean ± SEM, *n* = 3 experiments with independently cultured cells), while edaravone delayed H50 significantly to 145 ± 7, 165 ± 6, and 169 ± 9 h at concentrations of 2.2, 22, and 200 μmol/L, respectively (Table S2). In contrast, riluzole did not show significant protective effects on neurons against damage induced by oxidative stress at either level of AUC or H50 (Tables S3 and S4). These results suggest that the motor neuron damage induced by oxidative stress can recapitulate certain aspects of the ALS disease phenotype.

### 3.2 Involvement of Ferroptosis in Neuronal Damage Induced by Oxidative Stress

**FIGURE 2:**
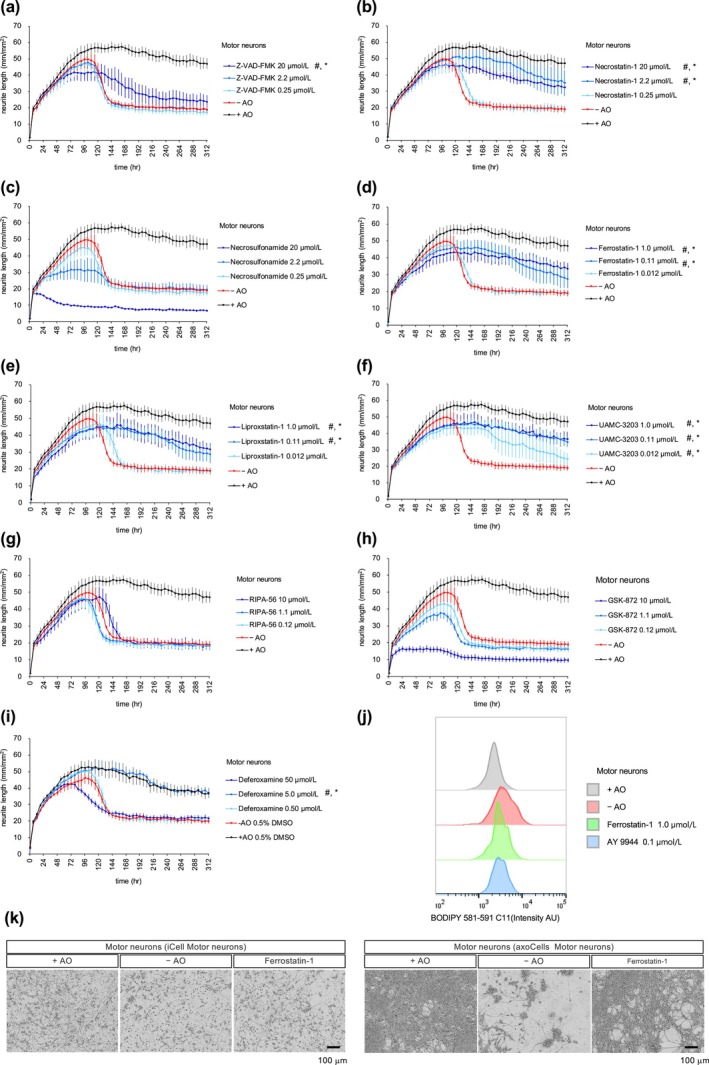
Protection of neurons from oxidative stress‐induced damage by ferroptosis inhibitors. (a–h) Time course changes in the total neurite length (mm/mm^2^) of motor neurons (iCell Motor neurons) cultured in DMEM/F12 with antioxidants (+AO) or without antioxidants (−AO). Z‐VAD‐FMK, necrostatin‐1, necrosulfonamide, ferrostatin‐1, liproxstatin‐1, UAMC‐3203, RIPA‐56, or GSK‐872 was added under the −AO condition. The lines represent the average of three experiments with independent cell culture preparations. Each experiment consists of six wells for the −AO and +AO groups, and two wells for compound treatment. Error bars indicate SEM. Significant effects increasing AUC and H50 are indicated by # and *, respectively, as determined using two‐way ANOVA followed by Dunnett's multiple comparison test. ^#^ *p* = 0.0061, Z‐VAD‐FMK at 20 μmol/L; *p* < 0.0001, necrostatin‐1 at 2.2 and 20 μmol/L; *p* < 0.0001, ferrostatin‐1 at 0.11 and 1.0 μmol/L; *p* < 0.0001, liproxstatin‐1 at 0.11 and 1.0 μmol/L; *p* < 0.0001, UAMC‐3203 at 0.012, 0.11, and 1.0 μmol/L. *p < 0.0001, Z‐VAD‐FMK at 20 μmol/L; *p* < 0.0001, necrostatin‐1 at 2.2 and 20 μmol/L; *p* < 0.0001, ferrostatin‐1 at 0.11 and 1.0 μmol/L; *p* < 0.0001, liproxstatin‐1 at 0.11 and 1.0 μmol/L; *p* < 0.0001, UAMC‐3203 at 0.012, 0.11, and 1.0 μmol/L. See Tables S5 and S6 for detailed statistical analysis. Note that these compound assays were conducted using the same plate sets, but the results are presented in separate graphs for clarity. Therefore, panels a–h share the same control data (+AO and −AO). (i) Time course of changes in the total neurite length (mm/mm^2^) of motor neurons cultured in +AO, −AO, or with deferoxamine under the −AO condition. The lines represent the average of three experiments with independent cell culture preparations. Error bars indicate SEM. Significant effects increasing AUC and H50 are indicated by # and *, respectively, as determined using two‐way ANOVA followed by Dunnett's multiple comparison test (# and *P < 0.0001 at 5.0 μmol/L). See Tables S7 and S8 for detailed statistical analysis. DMSO at 0.5% was included as the vehicle. (j) Flow cytometry analysis of BODIPY 581/591 C11 oxidation in motor neurons cultured in +AO, −AO, with ferrostatin‐1 (1.0 μmol/L) or AY 9944 (0.1 μmol/L) under the −AO condition for 5 days. (k) Representative images at 168 h of iCell Motor neurons and 312 h of axoCells Human iPSC‐Derived Motor Neurons cultured in DMEM/F12 with (+AO) or without AO (−AO), or with ferrostatin‐1 (1.0 μmol/L) under the −AO condition. Scale bar: 100 μm.

To understand the mode of neural cell death in the oxidative stress‐induced neuronal damage model, we treated motor neurons (iCell Motor neurons) with inhibitors of apoptosis (Z‐VAD‐FMK), necroptosis (necrostatin‐1 and necrosulfonamide), or ferroptosis (ferrostatin‐1, liproxstatin‐1, and UAMC‐3203) (Figure 2a–f, see also Figure 2k for representative images of motor neurons treated with ferrostatin‐1). While Z‐VAD‐FMK and necrosulfonamide showed limited or no neuroprotective effects under oxidative stress, necrostatin‐1 and ferroptosis inhibitors significantly inhibited neuronal damage induced by gradual oxidative stress at both AUC and H50 levels (Tables S5 and S6). Although necrostatin‐1 is known as an inhibitor of RIPK1, which is involved in necroptosis, it is reported to possess RIPK1‐independent activity in inhibiting ferroptosis (Yuk et al. 2021). To confirm that necrostatin‐1 exerts neuroprotective activity independent of the RIPK pathway, we examined whether RIPK inhibitors had neuroprotective activities. Neither RIPK1 inhibitor (RIPA‐56) nor RIPK3 inhibitor (GSK‐872) showed neuroprotective activity under oxidative stress (Figure 2g,h). To further confirm the involvement of ferroptosis, we examined the neuroprotective effects of deferoxamine (Figure 2i, Tables S7 and S8). Deferoxamine is an iron‐specific chelator that can inhibit ferroptosis. Neuroprotective effects of deferoxamine under oxidative stress further support the involvement of ferroptosis in neuronal death induced by gradual oxidative stress. To confirm the involvement of ferroptosis more directly, membrane lipid peroxidation was measured as a characteristic feature of ferroptosis using BODIPY 581/591 C11 fluorescent probe. The probe localizes to membranes in live cells and emits green fluorescent signal when oxidized. Increased signal was observed in neurons under oxidative stress and suppressed by the ferroptosis inhibitor (Figure 2j), indicating that this oxidative stress model accompanied by increased lipid peroxidation, which is a characteristic feature of ferroptosis.

We cultured motor neurons from a different source (axoCells Motor neurons) in DMEM/F12 without AO (−AO condition), treated them with ferrostatin‐1, and confirmed the induction of neuronal damage and the protective effects of ferrostatin‐1 in motor neurons from a distinct cell line, alongside iCell Motor neurons (Figure 2k). Furthermore, the neuronal damage by the −AO condition and neuroprotective effects of ferrostatin‐1 were also confirmed in NGN2‐induced neurons, FF‐1 and FF‐2 iNeurons (Figure S2a,b, Tables S11–S14). Increased lipid peroxidation and its suppression by ferrostatin‐1 were observed in FF‐2 iNeurons (Figure S2c), suggesting that oxidative stress‐induced neuronal damage in both motor neurons and NGN2‐induced neurons in a ferroptosis‐dependent manner.

### 3.3 Neuroprotective Effects of AY 9944

**FIGURE 3:**
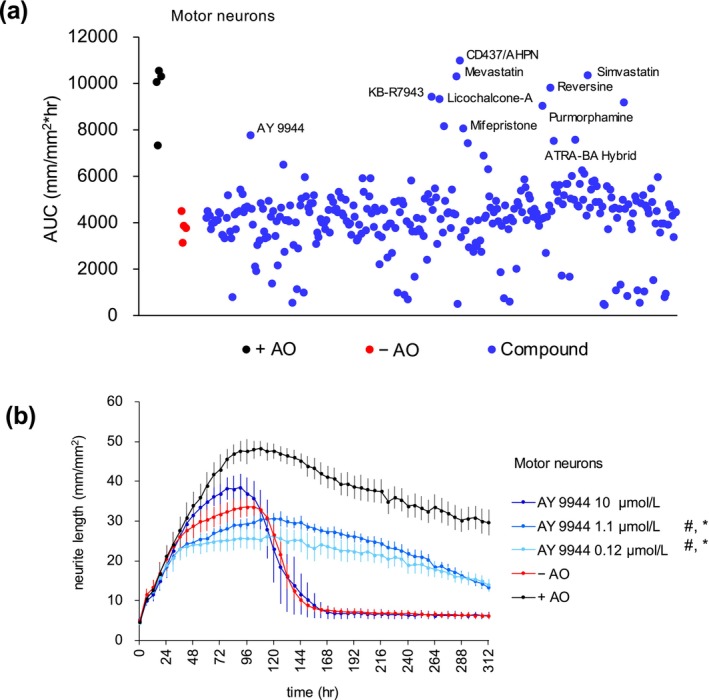
Focused small‐molecule screen and identification of AY 9944 as a neuroprotective compound. (a) Motor neurons (iCell Motor neurons) were cultured in DMEM/F12 media with antioxidants (+AO), without AO (−AO), or with compounds under the −AO condition. The area under the curve (AUC) of the total neurite length measurement graph (from Day 0 to Day 14) was calculated for each compound treatment and shown as a dot. (b) Time course changes in the total neurite length (mm/mm^2^) of motor neurons cultured in +AO, −AO, or with AY 9944 under the −AO condition. The lines represent the average of three experiments with independent cell culture preparations. Each experiment consists of 13–14 wells for the −AO and +AO groups, and four wells for compound treatment. Error bars indicate SEM. Significant effects increasing AUC and H50 are indicated by # and *, respectively, as determined using two‐way ANOVA followed by Dunnett's multiple comparison test (# and *p < 0.0001 at 0.12 and 1.1 μmol/L). See Tables S1 and S2 for detailed statistical analysis. Note that the experiments were conducted in the same plates with edaravone, and they include common control data from Figure 1g.

To identify compounds that inhibit oxidative stress‐induced neuronal damage, we screened the StemSelect library containing 303 small molecules that are pharmacologically active and structurally diverse. The area under the curve (AUC) of the total neurite length measurement graph was calculated for each compound treatment. The compounds that exhibited a value higher than twice the standard deviation from the mean of the AUC of the compound‐treated group (mean + 2 SD) were selected (Figure 3a). Some of the hits might have redox or radical scavenger activity (Hatano et al. 1988; Parthasarathy et al. 1994), and the hit compounds need to be carefully evaluated to determine whether they show effects due to physical properties or specific functions. Among the hit compounds, we focused on AY 9944, which is known to inhibit enzymes involved in sterol biosynthesis (Fernández et al. 2005). AY 9944 inhibited neuronal damage caused by gradual oxidative stress (Figure 3b). AY 9944 exhibited strong neuroprotective effects at a concentration of 1.1 μmol/L, while a higher (10 μmol/L) concentration of AY 9944 showed less pronounced effects (Figure 3b). It has been reported that AY 9944 inhibits several enzymes in the sterol biosynthesis pathway in the following order: DHCR7 > EBP > DHCR14 in human cells (Fernández et al. 2005). It has also been reported that 1 μmol/L of AY 9944 leads to the accumulation of 7‐dehydrocholesterol (7‐DHC), whereas higher concentrations (5, 10, or 20 μmol/L) of AY 9944 result in lesser or no accumulation of 7‐DHC, and instead lead to the accumulation of other intermediates (Fernández et al. 2005).

AY 9944 inhibited membrane lipid peroxidation under oxidative stress in both motor neurons and NGN2‐induced neurons (Figure 2j, Figure S2c), suggesting that AY 9944 inhibits neuronal damage by suppressing ferroptosis, consistent with recent reports of the anti‐ferroptotic activity of AY 9944 (Li et al. 2024; Yamada et al. 2024).

### 3.4 Involvement of Cholesterol Biosynthesis Pathway in Ferroptosis‐Dependent Neuronal Damage

**FIGURE 4:**
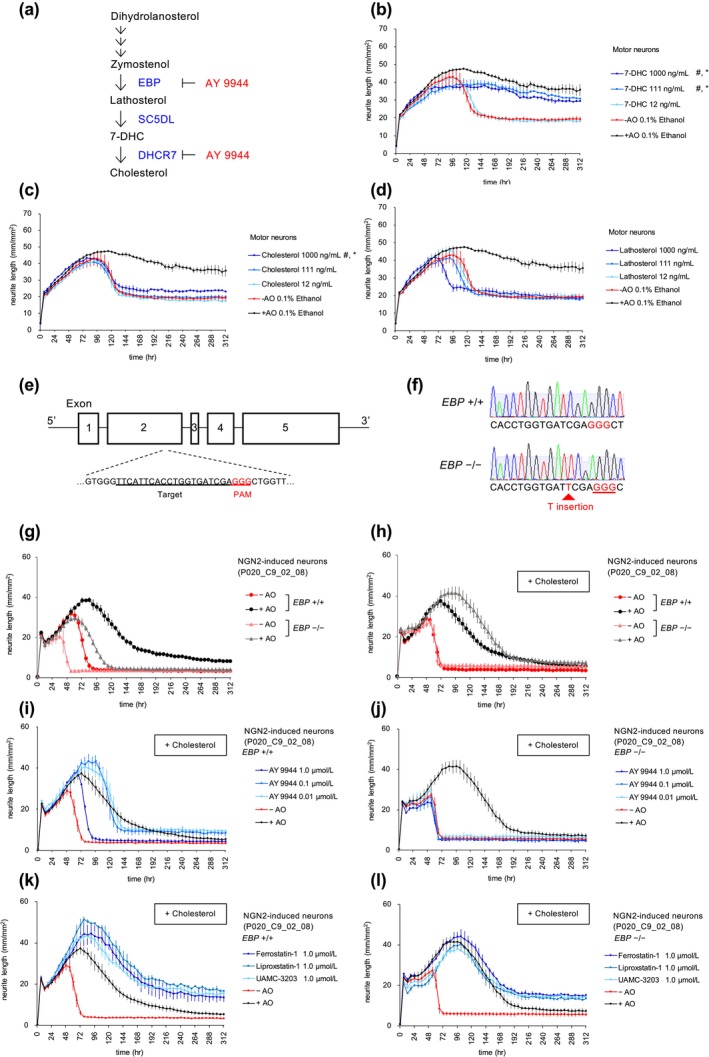
Involvement of cholesterol biosynthesis pathway in neuroprotection. (a) Schematic representation of the cholesterol biosynthetic steps. (b–d) Time course changes in the total neurite length (mm/mm^2^) of motor neurons (iCell Motor neurons) cultured in DMEM/F12 media with antioxidants (+AO), without AO (−AO), or with 7‐DHC, cholesterol, or lathosterol under the −AO condition. The lines represent the average of three experiments with independent cell culture preparations. Each experiment consists of three wells for the −AO and +AO groups, and two wells for compound treatment. Error bars indicate SEM. Significant effects increasing AUC and H50 are indicated by # and *, respectively, as determined using two‐way ANOVA followed by Dunnett's multiple comparison test. ^#^ *p* < 0.0001, 7‐DHC at 111 and 1000 ng/mL; *p* = 0.0071, cholesterol at 1000 ng/mL. *p < 0.0001, 7‐DHC at 111 and 1000 ng/mL; *p* = 0.0257, cholesterol at 1000 ng/mL. See Tables S9 and S10 for detailed statistical analysis. Note that these compound assays were conducted using the same plate sets, but the results are presented in separate graphs for clarity. Therefore, panels b–d share the same control data (+AO and −AO). (e) Diagram of the *EBP* gene. The target sequence of sgRNA is underlined, and the protospacer adjacent motif (PAM) sequence is indicated in red. (f) Genomic sequences of the *EBP* ^+/+^ or *EBP* ^−/−^ iPSC clones. The PAM sequence is indicated in red. A single nucleotide insertion in codon 79 in the *EBP* ^−/−^ iPSC clone resulted in a frameshift and early termination. (g) Time course changes in the total neurite length (mm/mm^2^) of the *EBP* ^+/+^ (closed circle) or *EBP* ^−/−^ (closed triangle) NGN2‐induced neurons. The lines represent the average of six wells for +AO and −AO conditions. Error bars show the standard deviation. (h–l) Time course changes in the total neurite length (mm/mm^2^) of the *EBP* ^+/+^ or *EBP* ^−/−^ NGN2‐induced neurons cultured with supplementation of 1.5 μg/mL cholesterol in the media. AY 9944 or ferroptosis inhibitors were added under the −AO condition. The lines represent the average of six wells for +AO and −AO conditions, and two wells for each compound treatment condition. Error bars show the standard deviation. Note that the control data is the same in (h), (i), and (k) for the *EBP* ^+/+^ neurons, and in (h), (j), and (l) for the *EBP* ^−/−^ neurons. Note that panels (h, i, k) and panels (h, j, l) share the same control data (+AO and −AO), because these assays were conducted using the same plate sets, but the results are presented in separate graphs for clarity.

It has recently been reported that 7‐DHC functions as a potent endogenous anti‐ferroptotic metabolite (Freitas et al. 2024; Li et al. 2024; Yamada et al. 2024). Considering that AY 9944 exhibited strong neuroprotective effects at 1.1 μmol/L, which is known to induce 7‐DHC accumulation by inhibition of DHCR7 in human cells (Fernández et al. 2005), our next objective was to examine whether 7‐DHC can protect motor neurons from ferroptosis‐dependent damage caused by oxidative stress. As expected, 7‐DHC inhibited neuronal damage caused by oxidative stress (Figure 4a,b). Meanwhile, cholesterol did not exhibit the neuroprotective effects at similar levels (Figure 4c). Furthermore, lathosterol did not demonstrate any notable neuroprotective activity (Figure 4d). Although lathosterol is expected to be metabolized into 7‐DHC in cells, the addition of lathosterol might not result in the accumulation of 7‐DHC in the cells.

### 3.5 Neuronal Damage Inhibition by AY 9944 Through Modulation of Sterol Biosynthesis Pathway

To gain further insight into the molecular mechanisms of AY 9944 in neuronal protection, we generated EBP knockout (*EBP* ^−/−^) NGN2‐induced neurons (Figure 4e,f). The *EBP* ^−/−^ neurons exhibited oxidative‐stress‐induced neuronal damage regardless of the presence or absence of cholesterol in the medium (Figure 4g,h). Without exogenously added cholesterol, the *EBP* ^−/−^ neurons showed poor growth compared to the *EBP* ^+/+^ control neurons. However, in the presence of AO with cholesterol supplementation, the *EBP* ^−/−^ neurons restored growth to the same level as the *EBP* ^+/+^ control neurons. Thus, the addition of cholesterol can differentiate the growth defects due to cholesterol depletion from neuronal damage induced by oxidative stress. Treatment with AY 9944 did not show neuroprotective activity against oxidative stress‐induced damage in the *EBP* ^−/−^ iPSC‐neurons, whereas ferroptosis inhibitors exhibited neuroprotection in the presence or absence of cholesterol (Figure 4i–l, Figure S3). These results indicate that AY 9944 exerts neuroprotective activity by modulating the sterol biosynthesis pathway.

## 4 Discussion

In this study, we established a neuronal damage model based on gradual oxidative stress by removing antioxidants from the culture medium using iPSC‐derived motor neurons and excitatory cortical neurons. The neuronal damage was induced by oxidative stress in DMEM/F12 but not in Neurobasal medium. DMEM/F12 medium contains ferrous sulfate and excitatory amino acids, whereas Neurobasal medium does not. These components could potentially influence cellular ferrous ion concentration and neuronal excitation, thus contributing to neuronal death induction through ferroptosis in combination with oxidative stress.

In the physiological brain, astrocytes can release GSH and other molecules to protect neurons from oxidative stress, and they also play a role in glutamate uptake and homeostasis, indirectly contributing to the protection of neurons from oxidative stress (Chen et al. 2020). In contrast, astrocytes undergo a transformation into reactive astrocytes and begin producing ROS and other molecules related to oxidative stress in neurodegenerative diseases (Chen et al. 2020). In this study, we tried to create an in vitro condition to replicate neurodegenerative diseases by removing antioxidants, in combination with specific medium conditions, using neuronal monoculture without astrocytes.

Neurons themselves also possess antioxidant systems and do not undergo immediate cell death when plated in media lacking antioxidants. Instead, they initially extend their neurites but subsequently experience a reduction in neurite length as they become unable to tolerate the oxidative stress, resulting in a gradual accumulation of oxidative stress levels. Hydrogen peroxide is frequently employed to induce oxidative stress, but there have been reported instances where ferroptosis inhibitors proved ineffective (Dixon et al. 2012; Wenz et al. 2018), likely due to acute oxidative stress induction or high treatment concentrations. In our model of gradual oxidative stress, we can expect to see different drug effects than in the acute stress model.

Edaravone, an approved drug for ALS, showed neuroprotective effects in our oxidative stress model, suggesting its potential usefulness as an in vitro ALS model. The reason why riluzole did not show effects in our model is uncertain; however, diverse actions of riluzole have been reported, including presynaptic reduction of glutamate release and increased glutamate uptake by astrocytes (Bellingham 2011; Carbone et al. 2012). It is possible that the effects of riluzole might not be detected in the presence of a high concentration (50 mmol/L) of glutamate in the medium and in the absence of astrocytes. Edaravone, a free radical scavenger, has been reported to suppress ferroptotic cell death in vitro (Homma et al. 2019). Pathological TDP‐43 protein aggregation is observed in 97% of ALS patients, serving as a hallmark of the disease (Ling et al. 2013). Zuo et al. (2021) reported the induction of TDP‐43 aggregation through hydrogen peroxide treatment in iPSC‐induced neurons. It is our future study to investigate whether our oxidative stress model is accompanied by TDP‐43 pathological features.

In our study, ferroptosis inhibitors protected neurons from damage induced by oxidative stress. Ferroptosis is gaining attention as a potential mechanism underlying neuronal loss in many neurodegenerative diseases, including AD and ALS (Wang, Wang, et al. 2022; Ou et al. 2022; Jakaria et al. 2021; Wang, Tomas, et al. 2022). The involvement of ferroptosis in our oxidative stress‐induced neuronal damage model aligns with previous findings connecting ferroptosis and neurodegeneration, as well as with the report by Tian et al. (2021), which highlights the implications of ferroptosis and oxidative stress in ALS. A recent clinical trial of deferiprone, an iron‐chelating agent, did not show beneficial effects in AD patients (Ayton et al. 2025). In our assay platform, deferoxamine showed neuroprotective effects at a certain concentration but exhibited toxic effects at higher concentrations (Figure 2i), suggesting the difficulty of handling the dosing of iron‐chelating agents. We hope that our assay platform can be utilized to find better compound for the treatment of neurodegenerative diseases.

The addition of 7‐DHC inhibited oxidative stress‐induced neuronal damage. It aligns with previous reports indicating the role of 7‐DHC as a potent endogenous anti‐ferroptotic metabolite (Freitas et al. 2024; Li et al. 2024; Yamada et al. 2024). Furthermore, the protection of neurons by AY 9944 was abolished by *EBP* knockout. These results suggest that the cholesterol biosynthesis pathway plays a role in the regulation of ferroptosis in neurons. Thus, the balance of cholesterol metabolism may play a role in the progression of neurodegenerative diseases. 7‐DHC levels are not significantly different in ALS patients (Abdel‐Khalik et al. 2017), and it might be difficult to state that the imbalance of cholesterol metabolism is directly involved in the onset of neurodegenerative diseases. However, modulating the metabolism pathway to boost the endogenous anti‐ferroptotic system might be a possible approach for drug development.

Given that 7‐DHC has anti‐ferroptotic activity, it is surprising that mevastatin and simvastatin were identified as hits in the screening. Both mevastatin and simvastatin are HMG‐CoA reductase inhibitors expected to reduce sterol synthesis, including 7‐DHC. We found that these statins protect motor neurons from oxidative stress, but not in induced excitatory cortical neurons (Figure 3a and our unpublished data). The metabolic states may differ between motor neurons and induced excitatory cortical neurons, and the balance might be shifted toward increased 7‐DHC levels in motor neurons through HMG‐CoA reductase inhibition, possibly due to complex regulation, including feedback regulation of the sterol synthesis pathway by cholesterol (Duan et al. 2022; Wassif et al. 2005). It has also been reported that statins, including mevastatin and simvastatin, induce antioxidant effects by modulating the Nrf2 signaling pathway (Mansouri et al. 2022). Metabolite analysis and pathway analysis will elucidate the mechanisms by which statins exert neuroprotective effects.

In conclusion, we have developed a method to induce neuronal damage through gradual oxidative stress. The observed neuronal damage was accompanied by lipid peroxidation, and it was inhibited by ferroptosis inhibitors. Neuroprotective effects by AY 9944 and 7‐DHC suggest possible involvement of the cholesterol biosynthesis pathway in modulating ferroptosis occurring in the oxidative stress‐induced neuronal injury. We expect that our model can contribute to drug development for ALS and other neurodegenerative diseases, wherein oxidative stress plays a central role in their etiology.

## Author Contributions

**Hayato Kobayashi:** conceptualization, investigation, writing – review and editing. **Hitoshi Suzuki‐Masuyama:** conceptualization, investigation, writing – review and editing. **Hirokazu Tanabe:** conceptualization, investigation, writing – review and editing. **Hiroshi Kato:** conceptualization, writing – review and editing, formal analysis. **Setsu Endoh‐Yamagami:** conceptualization, writing – original draft, writing – review and editing, formal analysis, investigation, supervision, project administration.

## Conflicts of Interest

All of the authors are employees of FUJIFILM Corporation. This work was supported by internal funding from FUJIFILM Corporation. Patents have been filed for a method to induce neuronal damage through oxidative stress by inventors Hayato Kobayashi, Hitoshi Suzuki‐Masuyama, Hirokazu Tanabe, Hiroshi Kato, and Setsu Endoh‐Yamagami, and for a method to study cholesterol pathway mutants by inventors Hayato Kobayashi and Setsu Endoh‐Yamagami.

## Peer Review

The peer review history for this article is available at https://www.webofscience.com/api/gateway/wos/peer‐review/10.1111/jnc.70246.

## Supporting information


**Appendix S1:** jnc70246‐sup‐0001‐AppendixS1.pdf.

## Data Availability

The data that support the findings of this study are available from the corresponding author upon reasonable request. A preprint of this article was posted on ResearchSquare; 09 July 2024; https://www.researchsquare.com/article/rs‐4602278/v1.
