# Supplementary material for: Neuronal Damage Induced by Gradual Oxidative Stress in iPSC‐Derived Neurons: Implications for Ferroptosis Involvement and ALS Drug Evaluation
Source: J Neurochem. 2025 Sep 28;169(10):e70246. doi: 10.1111/jnc.70246 (PMC12477418; doi:10.1111/jnc.70246)
Supplement: Supplementary file 1 — Appendix S1: jnc70246‐sup‐0001‐AppendixS1.pdf. [file JNC-169-0-s001.pdf]

Supporting Information

Neuronal damage induced by gradual oxidative stress in iPSC-derived neurons: Implications for ferroptosis involvement and ALS drug evaluation

Hayato Kobayashi, Hitoshi Suzuki-Masuyama, Hirokazu Tanabe, Hiroshi Kato, Setsu Endoh-Yamagami*

Bio Science & Engineering Laboratories, FUJIFILM Corporation, Kanagawa, Japan

Contact

*Correspondence: setsu.endo@fujifilm.com

Supplementary Figure 1

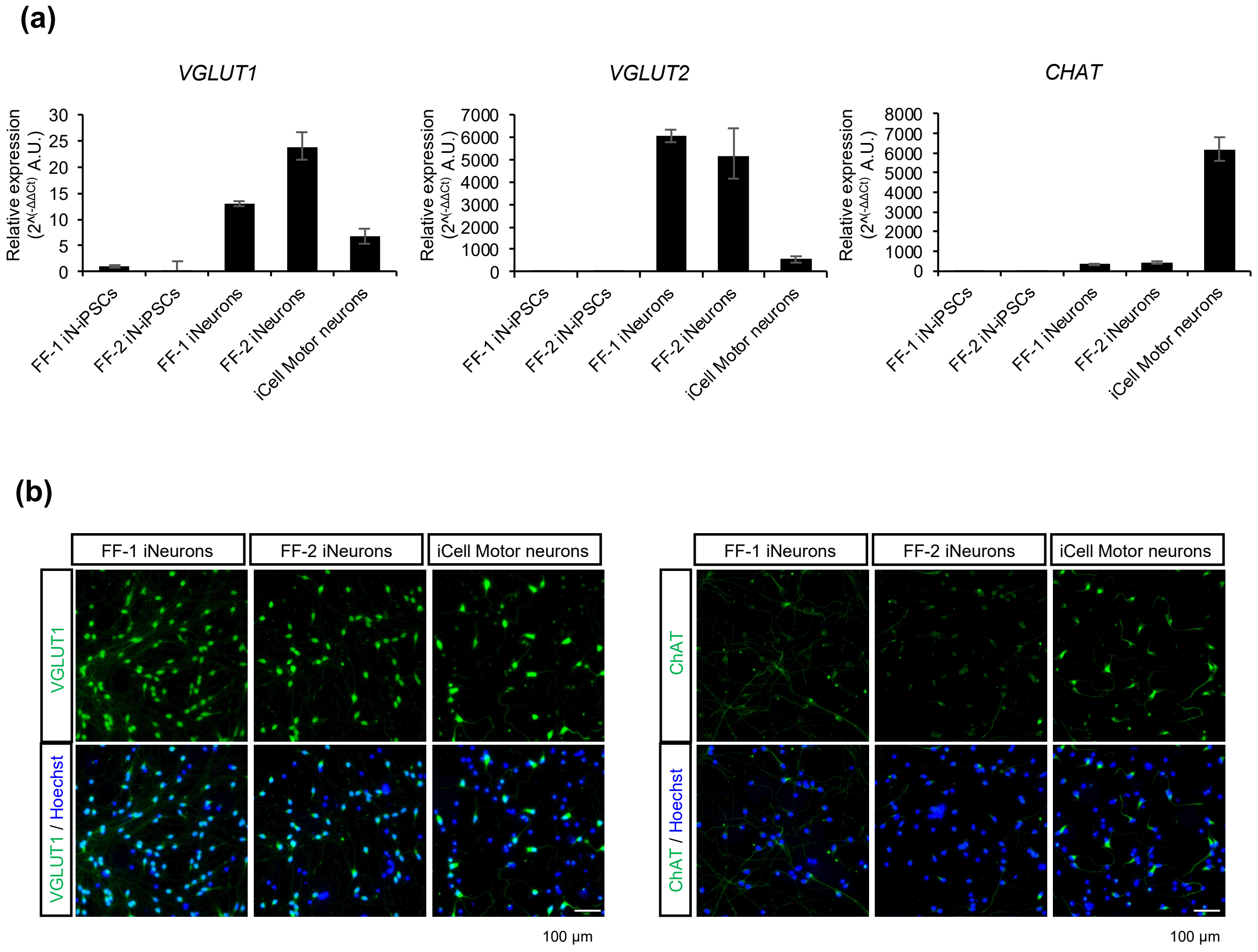

Supplementary Figure 1 Neuronal marker expression.
(**a**) Gene expression levels were examined for glutamatergic excitatory neuron markers (*VGLUT1* and *VGLUT2*) and a motor neuron marker (*CHAT*) in FF-1 iNeurons, FF-2 iNeurons, and iCell motor neurons cultured in DMEM/F12 with antioxidants for 3-4 days. Data indicates the average of 3 biological repeats with SEM. The gene expression levels were also examined in the iPSCs before differentiation into FF-1 and 2 iNeurons, FF-1 iN-iPSCs and FF-2 iN-iPSCs, respectively.
(**b**) Protein expression levels were confirmed by immunostaining for a glutamatergic excitatory neuron marker (VGLUT1) and a motor neuron marker (ChAT) in FF-1 iNeurons, FF-2 iNeurons, and iCell motor neurons cultured in DMEM/F12 with antioxidants for 3 days. Scale bar: 100 µm.

Supplementary Figure 2

(a)

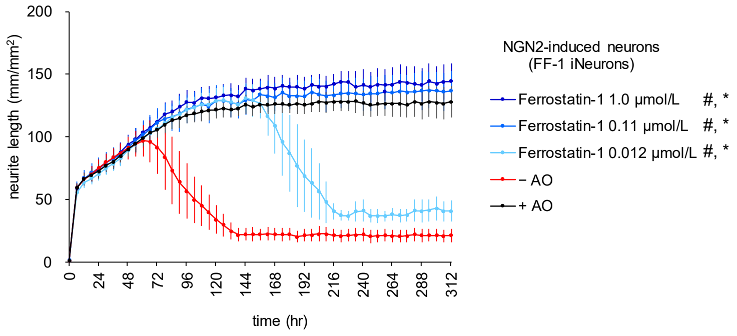

(b)

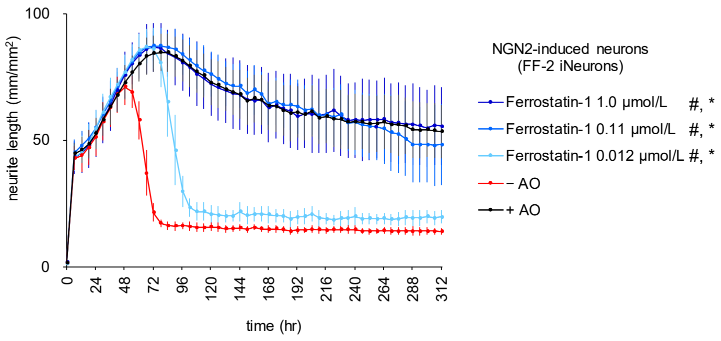

(c)

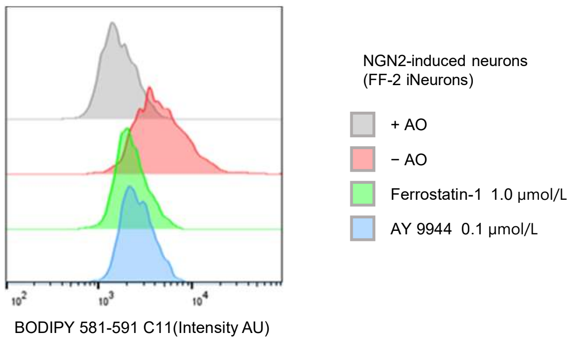

Supplementary Figure 2 Protection of neurons from oxidative stress-induced damage by ferroptosis inhibitors in NGN2-induced neurons.
(**a-b**) Time course changes in the total neurite length (mm/mm^2) of NGN-2 induced neurons, FF-1 iNeurons (a) and FF-2 iNeurons (b), cultured in DMEM/F12 with antioxidants (+ AO) or without antioxidants (− AO). Ferrostatin-1 was added under the − AO condition. The lines represent the average of the average of 3 experiments with independent cell culture preparations. Each experiment consists of 6 wells for the − AO and + AO groups, and 2 wells for compound treatment. Error bars indicate SEM. Significant effects increasing AUC and H50 are indicted by # and *, respectively (refer to Supplementary Tables 11-14 for detailed statistical analysis). (**c**) Flow cytometry analysis of BODIPYTM 581/591 C11 oxidation in FF-2 iNeurons cultured in + AO, – AO, with ferrostatin-1 (1.0 µmol/L) or AY 9944 (0.1 µmol/L) under the – AO condition for 2 days.

Supplementary Figure 3

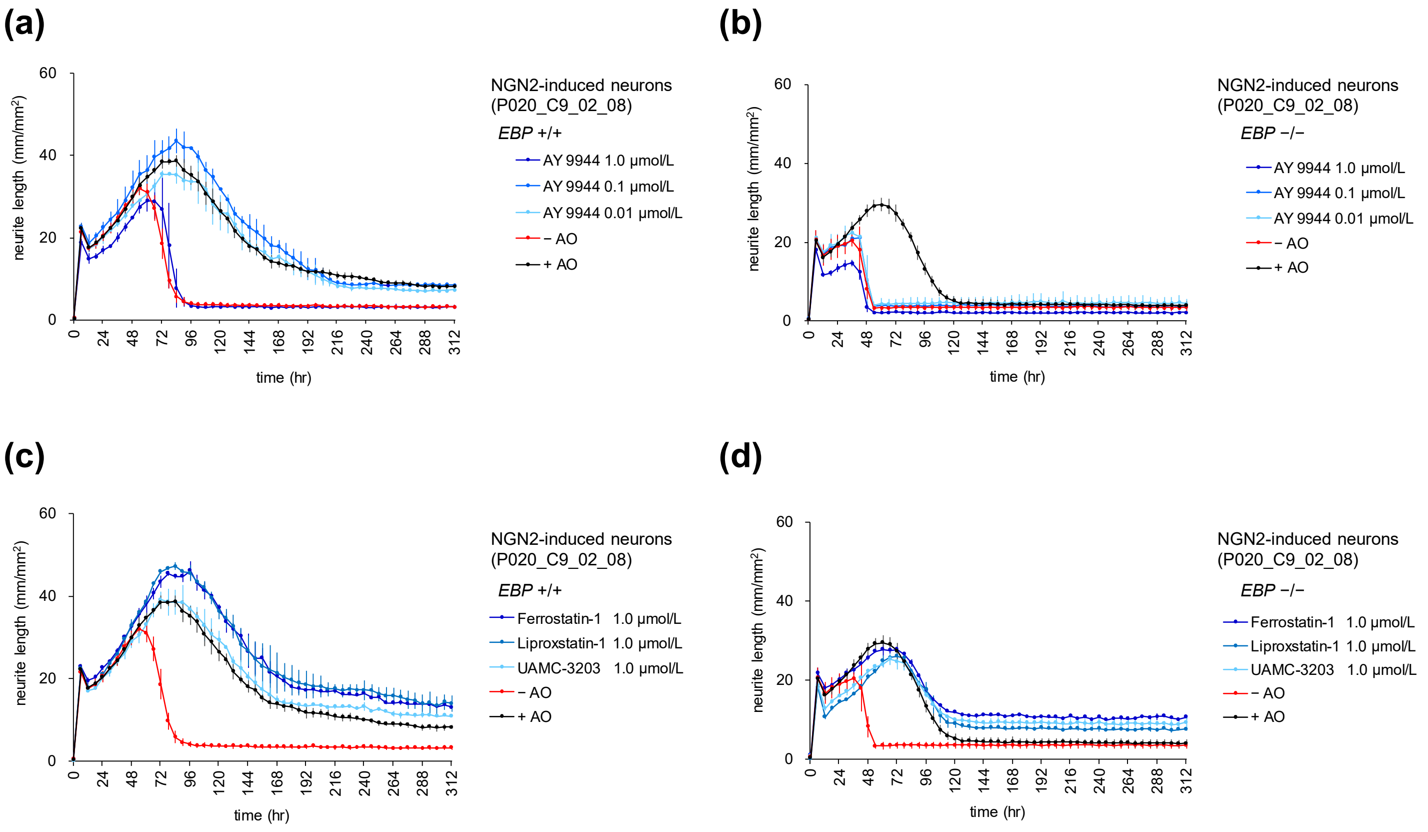

Supplementary Figure 3
Neurite length analysis of the *EBP* −/− NGN2-induced neurons.
(**a-d**) Time course changes of the total neurite length (mm/mm^2) of the *EBP* +/+ or *EBP* −/− NGN2-induced neurons treated with AY 9944 or ferroptosis inhibitors. The lines represent the average of 6 wells for + AO and − AO conditions, 1 well of AY 9944 (0.1 μmol/L) treatment in *EBP* −/− neurons, and 2 wells for the other compound treatment. Error bars show standard deviation. Note that panels (a, c) and panels (b, d) share the same control data (+ AO and – AO) of the *EBP* +/+ neurons and the *EBP* −/− neurons, respectively, with those data in Figure 4g, because these assays were conducted using the same plate sets, but the results are presented in separate graphs for clarity.

Supplementary Table 1
AUC data of neurite length analysis for Edaravone and AY 9944

				AUC (mm/mm^2*hr)	
**Treatment group**	**Exp. 1**	**Exp. 2**	**Exp. 3**	**Mean ± SEM**	***P* value**
- AO	4091 ± 743	4956 ± 947	5295 ± 562[‡]	4781 ± 358	NA
+ AO	10216 ± 1019	10832 ± 1263	12903 ± 1019	11317 ± 813	<0.0001
Edaravone 2.5 μmol/L	4464 ± 171	5209 ± 338	5420 ± 473	5031 ± 290	0.5711
Edaravone 22 μmol/L	5190 ± 242	5331 ± 638	6237 ± 451	5586 ± 328	0.0013 [#]
Edaravone 200 μmol/L	5474 ± 697	5013 ± 85	5379 ± 248	5289 ± 141	0.0656
AY9944 0.12 μmol/L	6077 ± 415	5723 ± 293	8003 ± 456	6601 ± 708	<0.0001 [#]
AY9944 1.1 μmol/L	7723 ± 623	6653 ± 995	7643 ± 756	7340 ± 344	<0.0001 [#]
AY9944 10 μmol/L	3837 ± 760	4549 ± 508	6426 ± 445	4937 ± 772	0.9209

Note: The average and standard deviation are shown for each experiment conducted using motor neurons. Each experiment consists of 14 wells for the − AO and + AO groups, and 4 wells for compound treatment samples with exception ‡ (13 wells). The mean and SEM of 3 experiments are indicated using cells prepared independently. The − AO and + AO groups are compared in 2-way ANOVA. The compound treatment effects are evaluated in 2 way-ANOVA followed by Dunnett's multiple comparison test in each compound, setting − AO as the control. #: Significant effects increasing AUC. NA: not applicable.

Supplementary Table 2
H50 data of neurite length analysis for Edaravone and AY 9944

				H50 (hr)	
**Treatment group**	**Exp. 1**	**Exp. 2**	**Exp. 3**	**Mean ± SEM**	***P* value**
- AO	134 ± 8	141 ± 10	124 ± 6[‡]	133 ± 5	NA
+ AO	312 ± 0	312 ± 2	312 ± 0	312 ± 0	ND
Edaravone 2.5 μmol/L	140 ± 6	159 ± 8	137 ± 9	145 ± 7	0.0001 *
Edaravone 22 μmol/L	164 ± 8	176 ± 8	155 ± 6	165 ± 6	<0.0001 *
Edaravone 200 μmol/L	173 ± 14	182 ± 9	152 ± 3	169 ± 9	<0.0001 *
AY9944 0.12 μmol/L	251 ± 70	213 ± 39	282 ± 23	249 ± 20	<0.0001 *
AY9944 1.1 μmol/L	296 ± 19	260 ± 19	273 ± 25	276 ± 11	<0.0001 *
AY9944 10 μmol/L	117 ± 6	123 ± 3	150 ± 11	130 ± 10	0.9679

Note: The average and standard deviation are shown for each experiment conducted using motor neurons. Each experiment consists of 14 wells for the − AO and + AO groups, and 4 wells for compound treatment samples with exception ‡ (13 wells). The mean and SEM of 3 experiments are indicated using cells prepared independently. The compound treatment effects are evaluated in 2 way-ANOVA followed by Dunnett's multiple comparison test in each compound, setting − AO as the control. *: Significant effects increasing H50. NA: not applicable. ND: not determined.

Supplementary Table 3
AUC data of neurite length analysis for Riluzole

	AUC (mm/mm^2*hr)					
**Treatment group**	**Exp. 1**	**Exp. 2**	**Exp. 3**	**Exp. 4**	**Mean ± SEM**	***P* value**
- AO	8730 ± 821	8242 ± 462	8268 ± 411	4091 ± 743	7333 ± 1086	NA
+ AO	12531 ± 1221	15874 ± 1257	15526 ± 1029	10216 ± 1019	13537 ± 1337	<0.0001
Riluzole 0.12 µmol/L	8818 ± 580	7187 ± 233	8141 ± 503	4750 ± 196	7224 ± 890	0.9937
Riluzole 1.1 µmol/L	9209 ± 175	8129 ± 525	8455 ± 120	4391 ± 852	7546 ± 1076	0.6214
Riluzole 10 µmol/L	9515 ± 0	8636 ± 413	9057 ± 128	4425 ± 406	7908 ± 1175	0.0532

Note: The average and standard deviation are shown for each experiment conducted using motor neurons. Each experiment is constituted of 6 wells for the − AO and + AO groups in Exp. 1-3 and 14 wells in Exp. 4, 2 wells for compound treatment samples in Exp. 1-3 and 4 wells in Exp. 4. The mean and SEM of 4 experiments are indicated using cells prepared independently. The − AO and + AO groups are compared in 2-way ANOVA. The compound treatment effects are evaluated in 2 way-ANOVA followed by Dunnett's multiple comparison test, setting − AO as the control. NA: not applicable.

Supplementary Table 4
H50 data of neurite length analysis for Riluzole

	H50 (hr)					
**Treatment group**	**Exp. 1**	**Exp. 2**	**Exp. 3**	**Exp. 4**	**Mean ± SEM**	***P* value**
- AO	142 ± 29	128 ± 12	127 ± 8	134 ± 8	133 ± 3	NA
+ AO	298 ± 34	312 ± 0	312 ± 0	312 ± 0	309 ± 4	ND
Riluzole 0.12 µmol/L	138 ± 0	120 ± 0	132 ± 0	143 ± 6	133 ± 5	0.9498
Riluzole 1.1 µmol/L	156 ± 0	141 ± 13	132 ± 0	138 ± 8	142 ± 5	0.1955
Riluzole 10 µmol/L	165 ± 13	132 ± 0	144 ± 0	135 ± 6	144 ± 7	0.1121

Note: The average and standard deviation are shown for each experiment conducted using motor neurons. Each experiment is constituted of 6 wells for the − AO and + AO groups in Exp. 1-3 and 14 wells in Exp. 4, 2 wells for compound treatment samples in Exp. 1-3 and 4 wells in Exp. 4. The mean and SEM of 4 experiments are indicated using cells prepared independently. The compound treatment effects are evaluated in 2 way-ANOVA followed by Dunnett's multiple comparison test, setting − AO as the control. NA: not applicable. ND: not determined.

Supplementary Table 5
AUC data of neurite length analysis for inhibitors of cell death

	AUC (mm/mm^2*hr)				
Treatment group	Exp. 1	Exp. 2	Exp. 3	Mean ± SEM	*P* value
- AO	7255 ± 355	8957 ± 365	9872 ± 621	8695 ± 767	NA
+ AO	14292 ± 544	14345 ± 1946	16548 ± 725	15062 ± 743	<0.0001
Z-VAD-FMK 0.25 µmol/L	6684 ± 368	8658 ± 236	8904 ± 66	8082 ± 703	0.2013
Z-VAD-FMK 2.2 µmol/L	7338 ± 554	8763 ± 119	9565 ± 182	8555 ± 651	0.9630
Z-VAD-FMK 20 µmol/L	7225 ± 252	9837 ± 2013	12399 ± 590	9820 ± 1494	0.0061 [#]
Necrostatin-1 0.25 µmol/L	6980 ± 634	9192 ± 16	9884 ± 556	8685 ± 876	>0.9999
Necrostatin-1 2.2 µmol/L	9873 ± 630	13704 ± 738	15871 ± 36	13149 ± 1754	<0.0001 [#]
Necrostatin-1 20 µmol/L	10603 ± 1074	11469 ± 692	13280 ± 3174	11784 ± 789	<0.0001 [#]
Necrosulfonamide 0.25 µmol/L	6656 ± 165	8360 ± 525	8617 ± 617	7878 ± 615	0.0314 [†]
Necrosulfonamide 2.2 µmol/L	5591 ± 137	7333 ± 432	9596 ± 194	7507 ± 1159	0.0013 [†]
Necrosulfonamide 20 µmol/L	2707 ± 21	2877 ± 11	3004 ± 23	2863 ± 86	<0.0001 [†]
Ferrostatin-1 0.012 µmol/L	7111 ± 138	9195 ± 1196	9334 ± 702	8547 ± 719	0.9747
Ferrostatin-1 0.11 µmol/L	9694 ± 91	10856 ± 1158	14558 ± 7	11703 ± 1467	<0.0001 [#]
Ferrostatin-1 1.0 µmol/L	10415 ± 255	10252 ± 444	14007 ± 1367	11558 ± 1225	<0.0001 [#]
Liproxstatin-1 0.012 µmol/L	7066 ± 205	8352 ± 486	10383 ± 305	8600 ± 966	0.9883
Liproxstatin-1 0.11 µmol/L	10024 ± 47	10672 ± 286	13533 ± 41	11410 ± 1078	<0.0001 [#]
Liproxstatin-1 1.0 µmol/L	10381 ± 177	9835 ± 383	14306 ± 1007	11507 ± 1408	<0.0001 [#]
UAMC-3203 0.012 µmol/L	8301 ± 381	10375 ± 1404	13380 ± 236	10685 ± 1474	<0.0001 [#]
UAMC-3203 0.11 µmol/L	11119 ± 711	11379 ± 506	14000 ± 1041	12166 ± 920	<0.0001 [#]
UAMC-3203 1.0 µmol/L	10930 ± 747	11245 ± 1462	14425 ± 319	12200 ± 1116	<0.0001 [#]
RIPA-56 0.12 µmol/L	7025 ± 541	7460 ± 252	9413 ± 1211	7966 ± 734	0.0328 [†]
RIPA-56 1.1 µmol/L	6354 ± 66	8297 ± 426	9407 ± 357	8019 ± 892	0.0518
RIPA-56 10 µmol/L	6696 ± 498	8460 ± 1071	10524 ± 469	8560 ± 1106	0.9397
GSK-872 0.12 µmol/L	6389 ± 12	7519 ± 310	8620 ± 147	7509 ± 644	<0.0001 [†]
GSK-872 1.1 µmol/L	5838 ± 383	7260 ± 1005	7451 ± 70	6850 ± 509	<0.0001 [†]
GSK-872 10 µmol/L	2898 ± 142	3819 ± 132	4635 ± 375	3784 ± 502	<0.0001 [†]

Note: The average and standard deviation are shown for each experiment conducted using motor neurons. Each experiment consists of 6 wells for the − AO and + AO groups, and 2 wells for compound treatment samples. The mean and SEM of 3 experiments are indicated using cells prepared independently. The − AO and + AO groups are compared in 2-way ANOVA. The compound treatment effects are evaluated in 2 way-ANOVA followed by Dunnett's multiple comparison test in each compound, setting - AO as the control. #: Significant effects increasing AUC. †: Significant effects decreasing AUC. NA: not applicable.

Supplementary Table 6
H50 data of neurite length analysis for inhibitors of cell death

	H50 (hr)				
**Treatment group**	**Exp. 1**	**Exp. 2**	**Exp. 3**	**Mean ± SEM**	***P* value**
- AO	148 ± 9	146 ± 11	136 ± 5	143 ± 4	NA
+ AO	312 ± 0	312 ± 0	312 ± 0	312 ± 0	ND
Z-VAD-FMK 0.25 µmol/L	141 ± 4	138 ± 0	129 ± 4	136 ± 4	0.9357
Z-VAD-FMK 2.2 µmol/L	144 ± 0	144 ± 8	135 ± 4	141 ± 3	0.9976
Z-VAD-FMK 20 µmol/L	168 ± 17	243 ± 98	297 ± 21	236 ± 37	<0.0001 *
Necrostatin-1 0.25 µmol/L	156 ± 8	156 ± 8	147 ± 4	153 ± 3	0.4504
Necrostatin-1 2.2 µmol/L	288 ± 34	312 ± 0	312 ± 0	304 ± 8	<0.0001 *
Necrostatin-1 20 µmol/L	312 ± 0	312 ± 0	276 ± 51	300 ± 12	<0.0001 *
Necrosulfonamide 0.25 µmol/L	144 ± 0	138 ± 17	126 ± 8	136 ± 5	0.2151
Necrosulfonamide 2.2 µmol/L	129 ± 4	123 ± 4	141 ± 4	131 ± 5	0.0226 †
Necrosulfonamide 20 µmol/L	ND	ND	ND	ND	ND
Ferrostatin-1 0.012 µmol/L	150 ± 0	165 ± 13	135 ± 13	150 ± 9	0.6322
Ferrostatin-1 0.11 µmol/L	261 ± 4	306 ± 8	312 ± 0	293 ± 16	<0.0001 *
Ferrostatin-1 1.0 µmol/L	312 ± 0	312 ± 0	312 ± 0	312 ± 0	<0.0001 *
Liproxstatin-1 0.012 µmol/L	156 ± 0	153 ± 4	156 ± 0	155 ± 1	0.1508
Liproxstatin-1 0.11 µmol/L	297 ± 21	312 ± 0	312 ± 0	307 ± 5	<0.0001 *
Liproxstatin-1 1.0 µmol/L	312 ± 0	273 ± 21	312 ± 0	299 ± 13	<0.0001 *
UAMC-3203 0.012 µmol/L	204 ± 17	285 ± 38	312 ± 0	267 ± 32	<0.0001 *
UAMC-3203 0.11 µmol/L	312 ± 0	312 ± 0	312 ± 0	312 ± 0	<0.0001 *
UAMC-3203 1.0 µmol/L	312 ± 0	309 ± 4	312 ± 0	311 ± 1	<0.0001 *
RIPA-56 0.12 µmol/L	132 ± 0	114 ± 8	120 ± 8	122 ± 5	<0.0001 †
RIPA-56 1.1 µmol/L	129 ± 4	132 ± 8	126 ± 0	129 ± 2	0.0035 †
RIPA-56 10 µmol/L	147 ± 4	156 ± 8	153 ± 13	152 ± 3	0.1050
GSK-872 0.12 µmol/L	138 ± 0	129 ± 4	129 ± 4	132 ± 3	0.0054 †
GSK-872 1.1 µmol/L	126 ± 8	123 ± 4	120 ± 0	123 ± 2	<0.0001 †
GSK-872 10 µmol/L	ND	ND	ND	ND	ND

Note: The average and standard deviation are shown for each experiment conducted using motor neurons. Each experiment consists of 6 wells for the − AO and + AO groups, and 2 wells for compound treatment samples. The mean and SEM of 3 experiments are indicated using cells prepared independently. The compound treatment effects are evaluated in 2 way-ANOVA followed by Dunnett's multiple comparison test in each compound, setting − AO as the control. *: Significant effects increasing H50. †: Significant effects decreasing H50. NA: not applicable. ND: not determined.

Supplementary Table 7
AUC data of neurite length analysis for Deferoxamine

	AUC (mm/mm^2*hr)				
**Treatment group**	**Exp. 1**	**Exp. 2**	**Exp. 3**	**Mean ± SEM**	***P* value**
-AO 0.5% DMSO	9646 ± 607	8025 ± 496	8400 ± 617	8690 ± 490	NA
+AO 0.5% DMSO	15177 ± 682	11769 ± 803	13134 ± 540	13360 ± 990	<0.0001
Deferoxamine 0.50 μmol/L	10015 ± 52	8593 ± 57	8495 ± 749	9034 ± 491	0.6147
Deferoxamine 5.0 μmol/L	13999 ± 701	11947 ± 762	13992 ± 180	13313 ± 683	<0.0001 #
Deferoxamine 50 μmol/L	9189 ± 899	8186 ± 424	8153 ± 223	8509 ± 340	0.9097

Note: The average and standard deviation are shown for each experiment conducted using motor neurons. Each experiment consists of 3 wells for the − AO and + AO groups, and 2 wells for compound treatment samples. DMSO was added to all samples at a final concentration of 0.5% as the vehicle. The mean and SEM of 3 experiments are indicated using cells prepared independently. The − AO and + AO groups are compared in 2-way ANOVA. The compound treatment effects are evaluated in 2 way-ANOVA followed by Dunnett's multiple comparison test, setting − AO as the control. #: Significant effects increasing AUC. NA: not applicable.

Supplementary Table 8
H50 data of neurite length analysis for Deferoxamine

	H50 (hr)				
**Treatment group**	**Exp. 1**	**Exp. 2**	**Exp. 3**	**Mean ± SEM**	***P* value**
-AO 0.5% DMSO	184 ± 17	136 ± 3	142 ± 3	154 ± 15	NA
+AO 0.5% DMSO	312 ± 0	312 ± 0	312 ± 0	312 ± 0	ND
Deferoxamine 0.50 μmol/L	174 ± 0	141 ± 13	138 ± 0	151 ± 12	0.9882
Deferoxamine 5.0 μmol/L	312 ± 0	312 ± 0	312 ± 0	312 ± 0	<0.0001 *
Deferoxamine 50 μmol/L	216 ± 59	129 ± 13	132 ± 0	159 ± 29	0.9499

Note: The average and standard deviation are shown for each experiment conducted using motor neurons. Each experiment consists of 3 wells for the − AO and + AO groups, and 2 wells for compound treatment samples. DMSO was added to all samples at a final concentration of 0.5% as the vehicle. The mean and SEM of 3 experiments are indicated using cells prepared independently. The compound treatment effects are evaluated in 2 way-ANOVA followed by Dunnett's multiple comparison test, setting − AO as the control. *: Significant effects increasing H50. NA: not applicable. ND: not determined.

Supplementary Table 9
AUC data of neurite length analysis for sterols

	AUC (mm/mm^2*hr)				
**Treatment group**	**Exp. 1**	**Exp. 2**	**Exp. 3**	**Mean ± SEM**	***P* value**
-AO 0.1% Ethanol	7271 ± 755	8734 ± 620	7860 ± 313	7955 ± 425	NA
+AO 0.1% Ethanol	11512 ± 145	12743 ± 949	12139 ± 527	12131 ± 355	<0.0001
7-DHC 12 ng/mL	8100 ± 229	7671 ± 611	7843 ± 23	7871 ± 125	0.9933
7-DHC 111 ng/mL	10664 ± 1503	9762 ± 168	11126 ± 384	10517 ± 401	<0.0001 #
7-DHC 1000 ng/mL	10468 ± 85	9960 ± 467	10582 ± 518	10337 ± 191	<0.0001 #
Cholesterol 12 ng/mL	7130 ± 225	7941 ± 264	7553 ± 83	7541 ± 234	0.3718
Cholesterol 111 ng/mL	7827 ± 1	8160 ± 233	7781 ± 87	7923 ± 119	0.9991
Cholesterol 1000 ng/mL	9046 ± 69	8637 ± 1195	9118 ± 44	8934 ± 150	0.0071 #
Lathosterol 12 ng/mL	7778 ± 708	8873 ± 1584	7774 ± 131	8142 ± 366	0.8947
Lathosterol 111 ng/mL	6963 ± 61	8131 ± 308	7498 ± 204	7531 ± 338	0.4324
Lathosterol 1000 ng/mL	7980 ± 292	7980 ± 320	7039 ± 144	7666 ± 314	0.7097

Note: The average and standard deviation are shown for each experiment conducted using motor neurons. Each experiment consists of 3 wells for the − AO and + AO groups, and 2 wells for compound treatment samples. Ethanol was added to all samples at a final concentration of 0.1% as the vehicle. The mean and SEM of 3 experiments are indicated using cells prepared independently. The − AO and + AO groups are compared in 2-way ANOVA. The compound treatment effects are evaluated in 2 way-ANOVA followed by Dunnett's multiple comparison test, setting − AO as the control. #: Significant effects increasing AUC. NA: not applicable.

Supplementary Table 10
H50 data of neurite length analysis for sterols

	H50 (hr)				
**Treatment group**	**Exp. 1**	**Exp. 2**	**Exp. 3**	**Mean ± SEM**	***P* value**
-AO 0.1% Ethanol	126 ± 6	150 ± 10	128 ± 3	135 ± 8	NA
+AO 0.1% Ethanol	312 ± 0	312 ± 0	312 ± 0	312 ± 0	ND
7-DHC 12 ng/mL	138 ± 0	144 ± 17	141 ± 4	141 ± 2	0.7037
7-DHC 111 ng/mL	312 ± 0	312 ± 0	312 ± 0	312 ± 0	<0.0001 *
7-DHC 1000 ng/mL	312 ± 0	288 ± 34	312 ± 0	304 ± 8	<0.0001 *
Cholesterol 12 ng/mL	120 ± 0	132 ± 0	129 ± 4	127 ± 4	0.8126
Cholesterol 111 ng/mL	129 ± 4	135 ± 13	126 ± 0	130 ± 3	0.9469
Cholesterol 1000 ng/mL	207 ± 21	138 ± 25	147 ± 4	164 ± 22	0.0257 *
Lathosterol 12 ng/mL	129 ± 13	162 ± 68	123 ± 13	138 ± 12	0.9851
Lathosterol 111 ng/mL	111 ± 4	126 ± 8	123 ± 4	120 ± 5	0.4734
Lathosterol 1000 ng/mL	132 ± 51	129 ± 4	93 ± 4	118 ± 13	0.3721

Note: The average and standard deviation are shown for each experiment conducted using motor neurons. Each experiment consists of 3 wells for the − AO and + AO groups, and 2 wells for compound treatment samples. Ethanol was added to all samples at a final concentration of 0.1% as the vehicle. The mean and SEM of 3 experiments are indicated using cells prepared independently. The compound treatment effects are evaluated in 2 way-ANOVA followed by Dunnett's multiple comparison test, setting − AO as the control. *: Significant effects increasing H50. NA: not applicable. ND: not determined.

Supplementary Table 11
AUC data of neurite length analysis for Ferrostatin-1 in FF-1 iNeurons

	AUC (mm/mm^2*hr)				
**Treatment group**	**Exp. 1**	**Exp. 2**	**Exp. 3**	**Mean ± SEM**	***P* value**
- AO	15270 ± 4971	7620 ± 806	15622 ± 3598	12837 ± 2611	NA
+ AO	36464 ± 2209	30985 ± 1963	39145 ± 1716	35531 ± 2401	<0.0001
Ferrostatin-1 0.012 µmol/L	22723 ± 1533	20460 ± 1631	30647 ± 1471	24610 ± 3088	<0.0001 #
Ferrostatin-1 0.11 µmol/L	40088 ± 1003	30003 ± 1792	41828 ± 1173	37306 ± 3686	<0.0001 #
Ferrostatin-1 1.0 µmol/L	40556 ± 567	32957 ± 2241	42990 ± 662	38834 ± 3021	<0.0001 #

Note: The average and standard deviation are shown for each experiment conducted using NGN2-induced neurons, FF-1 iNeurons. Each experiment consists of 6 wells for the − AO and + AO groups, and 2 wells for compound treatment samples. The mean and SEM of 3 experiments are indicated using cells prepared independently. The − AO and + AO groups are compared in 2-way ANOVA. The compound treatment effects are evaluated in 2 way-ANOVA followed by Dunnett's multiple comparison test, setting − AO as the control. #: Significant effects increasing AUC. NA: not applicable.

Supplementary Table 12
H50 data of neurite length analysis for Ferrostatin-1 in FF-1 iNeurons

	H50 (hr)				
**Treatment group**	**Exp. 1**	**Exp. 2**	**Exp. 3**	**Mean ± SEM**	***P* value**
- AO	108 ± 22	78 ± 8	106 ± 17	97 ± 10	NA
+ AO	312 ± 0	312 ± 0	312 ± 0	312 ± 0	ND
Ferrostatin-1 0.012 µmol/L	177 ± 4	195 ± 13	216 ± 8	196 ± 11	<0.0001 *
Ferrostatin-1 0.11 µmol/L	312 ± 0	312 ± 0	312 ± 0	312 ± 0	<0.0001 *
Ferrostatin-1 1.0 µmol/L	312 ± 0	312 ± 0	312 ± 0	312 ± 0	<0.0001 *

Note: The average and standard deviation are shown for each experiment conducted using NGN2-induced neurons, FF-1 iNeurons. Each experiment consists of 6 wells for the − AO and + AO groups, and 2 wells for compound treatment samples. The mean and SEM of 3 experiments are indicated using cells prepared independently. The − AO and + AO groups are compared in 2-way ANOVA. The compound treatment effects are evaluated in 2 way-ANOVA followed by Dunnett's multiple comparison test, setting − AO as the control. *: Significant effects increasing H50. NA: not applicable. ND: not determined.

Supplementary Table 13
AUC data of neurite length analysis for Ferrostatin-1 in FF-2 iNeurons

	AUC (mm/mm^2*hr)				
**Treatment group**	**Exp. 1**	**Exp. 2**	**Exp. 3**	**Mean ± SEM**	***P* value**
- AO	7054 ± 935	6165 ± 340	8714 ± 1102	7311 ± 747	NA
+ AO	16808 ± 1617	17320 ± 1851	25491 ± 1189	19873 ± 2813	<0.0001
Ferrostatin-1 0.012 µmol/L	8787 ± 252	9460 ± 455	13071 ± 818	10439 ± 1330	0.0202 #
Ferrostatin-1 0.11 µmol/L	15551 ± 7	19067 ± 279	25811 ± 1348	20143 ± 3010	<0.0001 #
Ferrostatin-1 1.0 µmol/L	14859 ± 6	17486 ± 890	27824 ± 3636	20056 ± 3957	<0.0001 #

Note: The average and standard deviation are shown for each experiment conducted using NGN2-induced neurons, FF-2 iNeurons. Each experiment consists of 6 wells for the − AO and + AO groups, and 2 wells for compound treatment samples. The mean and SEM of 3 experiments are indicated using cells prepared independently. The − AO and + AO groups are compared in 2-way ANOVA. The compound treatment effects are evaluated in 2 way-ANOVA followed by Dunnett's multiple comparison test, setting − AO as the control. #: Significant effects increasing AUC. NA: not applicable.

Supplementary Table 14
H50 data of neurite length analysis for Ferrostatin-1 in FF-2 iNeurons

	H50 (hr)				
**Treatment group**	**Exp. 1**	**Exp. 2**	**Exp. 3**	**Mean ± SEM**	***P* value**
- AO	72 ± 4	66 ± 0	67 ± 5	68 ± 2	NA
+ AO	299 ± 24	312 ± 0	312 ± 0	308 ± 4	ND
Ferrostatin-1 0.012 µmol/L	90 ± 0	102 ± 0	99 ± 4	97 ± 4	0.0009 *
Ferrostatin-1 0.11 µmol/L	255 ± 13	312 ± 0	312 ± 0	293 ± 19	<0.0001 *
Ferrostatin-1 1.0 µmol/L	273 ± 38	312 ± 0	312 ± 0	299 ± 13	<0.0001 *

Note: The average and standard deviation are shown for each experiment conducted using NGN2-induced neurons, FF-2 iNeurons. Each experiment consists of 6 wells for the − AO and + AO groups, and 2 wells for compound treatment samples. The mean and SEM of 3 experiments are indicated using cells prepared independently. The compound treatment effects are evaluated in 2 way-ANOVA followed by Dunnett's multiple comparison test, setting − AO as the control. *: Significant effects increasing H50. NA: not applicable. ND: not determined.

Supplementary Materials and Methods

RNA extraction and qRT-PCR

Total RNA was extracted by using QIAseq UPX Cell Lysis Kit (Qiagen) with reference to the manufacturer's instructions. qRT-PCR was conducted using the QuantiTect SYBR Green RT-PCR Kit (Qiagen), following manufacturer' protocol. The following primers were used: GAPDH, sense: 5'-gtcagtggtggacctgacct-3', antisense: 5'-tgctgtagccaaattcgttg-3'; VGLUT1, sense: 5'-acacggctcctttttctgg-3', antisense: 5'-cctcacgaagatgacacagc-3'; VGLUT2, sense: 5'-tggggctacatcatcactca-3', antisense: 5'-aggtcacaccctcaacaagtc-3'; CHAT, sense: 5'-ggctcagaacagcagcatc-3', antisense: 5'-ggtggagtctttcacgagga-3'). qRT-PCR was conducted using the CFX384 Touch Real-Time PCR Detection System (Bio-Rad). The data were analyzed using the delta-delta Ct method with GAPDH as the endogenous control.

Immunostaining

Cells were fixed by adding formalin solution (Fujifilm Wako) and incubating at room temperature for 30 minutes. After washing 3 times with PBS (−), the cells were permeabilized by treating with a blocking solution containing 1% BSA (Sigma Aldrich) and 0.1% Triton X-100 (BioVision) in PBS (−) for 30 minutes. Primary antibodies were diluted in blocking solution as follows: anti-VGLUT1, rabbit, 1:200, Proteintech; anti-CHAT, rabbit, 1:200, Proteintech. Cells were incubated with primary antibodies overnight at 4° C. Secondary antibodies were diluted in PBS (−) containing 1% BSA (Sigma Aldrich) as follows: anti-rabbit Alexa Fluor 488, 1:1000, Thermo Fisher Scientific and Hoechst 33342, 1:1000, Dojindo. Following 3-time washes with PBS (−), cells were incubated with secondary antibodies for 1 hour at room temperature. After additional 3-time washes with PBS (−), images were captured using an ECLIPSE Ti microscope (Nikon).
